# Supplementary material for: Renal tubular function and morphology revealed in kidney without labeling using three-dimensional dynamic optical coherence tomography
Source: Sci Rep. 2023 Sep 15;13:15324. doi: 10.1038/s41598-023-42559-3 (PMC10504276; doi:10.1038/s41598-023-42559-3)
Supplement: Supplementary file 1 — Supplementary Figure 1. [file 41598_2023_42559_MOESM1_ESM.pdf]

Supplementary material

Renal tubular function and morphology revealed in kidney without labeling using three-dimensional dynamic optical coherence tomography

Pradipta Mukherjee[1], Shinichi Fukuda[2, 3, *], Donny Lukmanto[2], Thi Hang Tran[2, 4, 5], Kosuke Okada[6], Shuichi Makita[1], Ibrahim Abd El-Sadek[1, 7], Yiheng Lim[1], and Yoshiaki Yasuno[1, *]

Figure S1 summarizes LIV projection images of sixteen distinct kidney samples obtained from normal mice. The LIV images consistently reveal prominent pipe-like structures, representing the renal tubules of the kidneys, across all the samples.

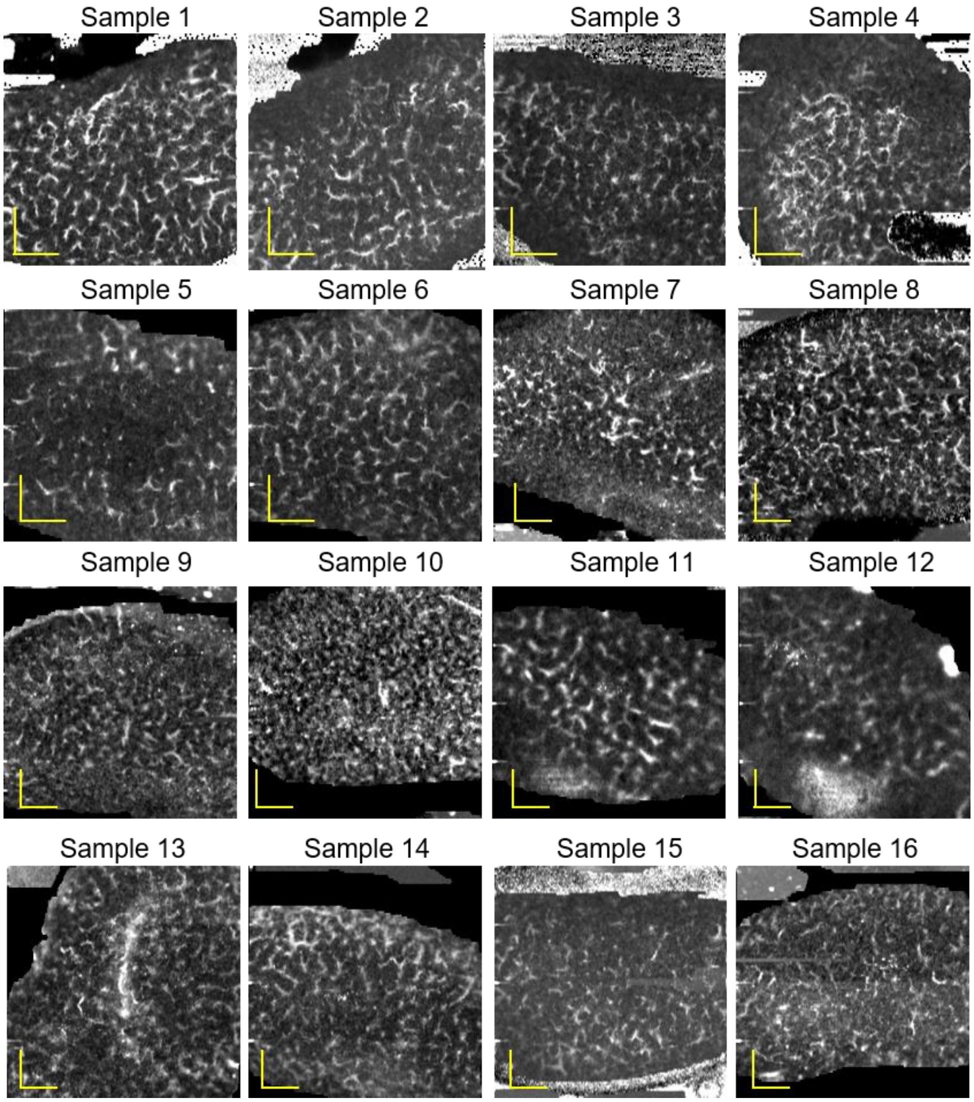

Figure S1: LIV projection images of sixteen different healthy mouse kidneys at initial time point (after sacrification).
